# Preoperative Versus Postoperative Transarterial Chemoembolization on Prognosis of Large Hepatocellular Carcinoma

**DOI:** 10.7150/jca.55806

**Published:** 2021-08-28

**Authors:** Xiaohui Wang, Yu Yuan, Juncheng Wang, Zishan Liu, Minshan Chen, Qunfang Zhou, Zhongguo Zhou

**Affiliations:** 1 Collaborative Innovation Center for Cancer Medicine, State Key Laboratory of Oncology in South China, Sun Yat-Sen University Cancer Center, Guangzhou, Guangdong, 510060, P. R. China; 2 Department of Liver Surgery, Sun Yat-Sen University Cancer Center, Guangzhou, Guangdong, 510060, P. R. China.; 3 Department of Pulmonary and Critical Care Medicine, The Second Xiangya Hospital, Central South University, 139 Renmin Middle Road, Changsha, Hunan 410011, China.; 4 Department of Minimally Invasive Interventional Radiology, and Department of Radiology, the Second Affiliated Hospital of Guangzhou Medical University, Guangzhou, 510260, China.

**Keywords:** Large hepatocellular carcinoma, Transarterial chemoembolization, Liver resection, Prognosis, Propensity score matching

## Abstract

**Background:** Transarterial chemoembolization (TACE) has proven to be an effective adjuvant therapy with liver resection (LR) to treat patients with hepatocellular carcinoma (HCC). The aim of this study was to evaluate outcomes in patients with HCC larger than 5 cm, comparing those who had TACE before LR to those who had TACE after LR.

**Materials and methods:** A total of 320 consecutive patients who underwent LR in combination with TACE for HCC larger than 5 cm from January 2009 to December 2014 were enrolled in study. Patients were divided into two groups: preoperative TACE group (n=199) and postoperative TACE group (n=121). Overall survival (OS) and recurrence-free survival (RFS) of patients were compared between preoperative TACE and postoperative TACE groups by propensity score-matching (PSM). We determined prognostic factors for recurrence and death using multivariate cox regression analysis.

**Results:** Among the 320 patients, the median age was 48 (range, 18 to 75) years, and 285 (89.1%) patients were male. During the follow- up period, 88 (44.2%) patients in the preoperative TACE group and 69 (57.0%) patients in the postoperative TACE group died. Before PSM, both OS and RFS were significantly longer in the preoperative TACE group than those in the postoperative TACE group (*P*=0.001 and *P*<0.001, respectively). After PSM, compared to those received postoperative TACE, patients with preoperative TACE had significantly better OS (Hazard ratio [HR]=1.92; 95% confidence interval [CI], 1.22-3.02; *P*=0.005) and RFS (HR=1.64; 95% CI, 1.16-2.32; *P*=0.005).

**Conclusions:** Patients with large HCC undergoing LR appear to derive greater disease control and survival benefit from a single preoperative TACE treatment than from postoperative TACE.

## Introduction

Hepatocellular carcinoma (HCC) is the sixth most common form of cancer and the fourth most common cause of cancer-related death globally 1. More than half of all HCC cases are diagnosed in China, and many patients are not diagnosed until their tumors have grown to be large (>5 cm) or huge (≥10 cm) 2. Currently, liver resection (LR) is the preferred method of treatment for patients with HCC 3. Other treatments that are available include radiofrequency ablation (RFA), microwave ablation (MWA), cryoablation, and transarterial chemoembolization (TACE). Unfortunately, the high postsurgical recurrence rate of large HCC (>5 cm) has compromised the long-term survival 4, 5.

TACE is typically used as a locoregional or palliative therapy for HCC 6-8. However, TACE has been used as a neoadjuvant therapy for large HCC prior to LR 7. In addition, a systematic review has demonstrated that TACE can be combined with other modalities to improve the resectability rate for HCC 9. Large HCC tumors are usually rich in neovascularization and frequently associated with micrometastases 10. TACE effectively blocks tumor-feeding vessels via the hepatic artery, thereby killing foci of tumor in the areas treated 9. Preoperative TACE therapy may also permit curative resection in some patients with large HCC who were not initially deemed to have resectable tumors 10, 11. However, preoperative TACE therapy remains controversial. Some studies have shown that preoperative TACE does not improve the long-time survival 12, 13. On the contrary, the use of preoperative TACE followed by LR has been shown to improve survival outcomes for some patients 14.

TACE has also been evaluated as a postoperative adjuvant therapy. Several studies have reported that postoperative TACE improved the survival of patients with HCC with portal vein tumor thrombus 15-17. Another report focusing on patients with hepatitis B virus (HBV)-related HCC showed that those who received TACE after LR had significantly longer recurrence-free survival (RFS) and overall survival (OS) than those who had LR alone 18. In that study, RFS was 25.7 months longer for those who received TACE after LR compared to those who did not. On subgroup analysis, postoperative TACE even provided clinical benefit to the patients in that study who had characteristics typically associated with poor prognosis, including young age, high alpha-fetoprotein (AFP) levels, cirrhosis, and other factors suggesting a high risk of recurrence (a single tumor with microvascular invasion, or multiple tumors) 19.

It remains unclear whether preoperative or postoperative TACE is more effective in prolonging survival and preventing recurrence in patients with large HCC who undergo LR. The aim of this study was to compare the OS and RFS rates of patients with large HCC (>5cm) who had TACE before LR and those who had TACE after LR.

## Materials and methods

### Patient Cohort

We retrospectively reviewed data on consecutive patients diagnosed with large HCC (>5 cm) who had undergone liver resection at Sun Yat-sen University Cancer Center (SYSUCC) between January 2009 and December 2014. Patients were included if they met the following criteria: (1) 18 to 75 years of age; (2) Child-Pugh grade A or B liver function; (3) patients with portal vein tumor thrombosis (PVTT) were limited in the right or the left branches of portal vein; (4) patients with multi-tumors less 3, and the maximum size of the satellite was less than 3cm; (5) histopathologically confirmed HCC; (6) tumor diameter greater than 5 cm evaluated by imaging data (computed tomography [CT], magnetic resonance imaging [MRI]); (7) absence of extrahepatic metastases; (8) curative resection; (9) TACE received within 3 months before or 3 months after LR. Patients were excluded from the analysis if they: (1) had recurrent HCC; (2) non-R0 liver resection; (3) received previous systemic chemotherapy, targeted therapy with Solafenib, or RFA for HCC; (4) lost to follow-up within 90 days after LR; (5) incomplete clinical data; (6) received both preoperative and postoperative TACE therapy.

**Figure 1:**
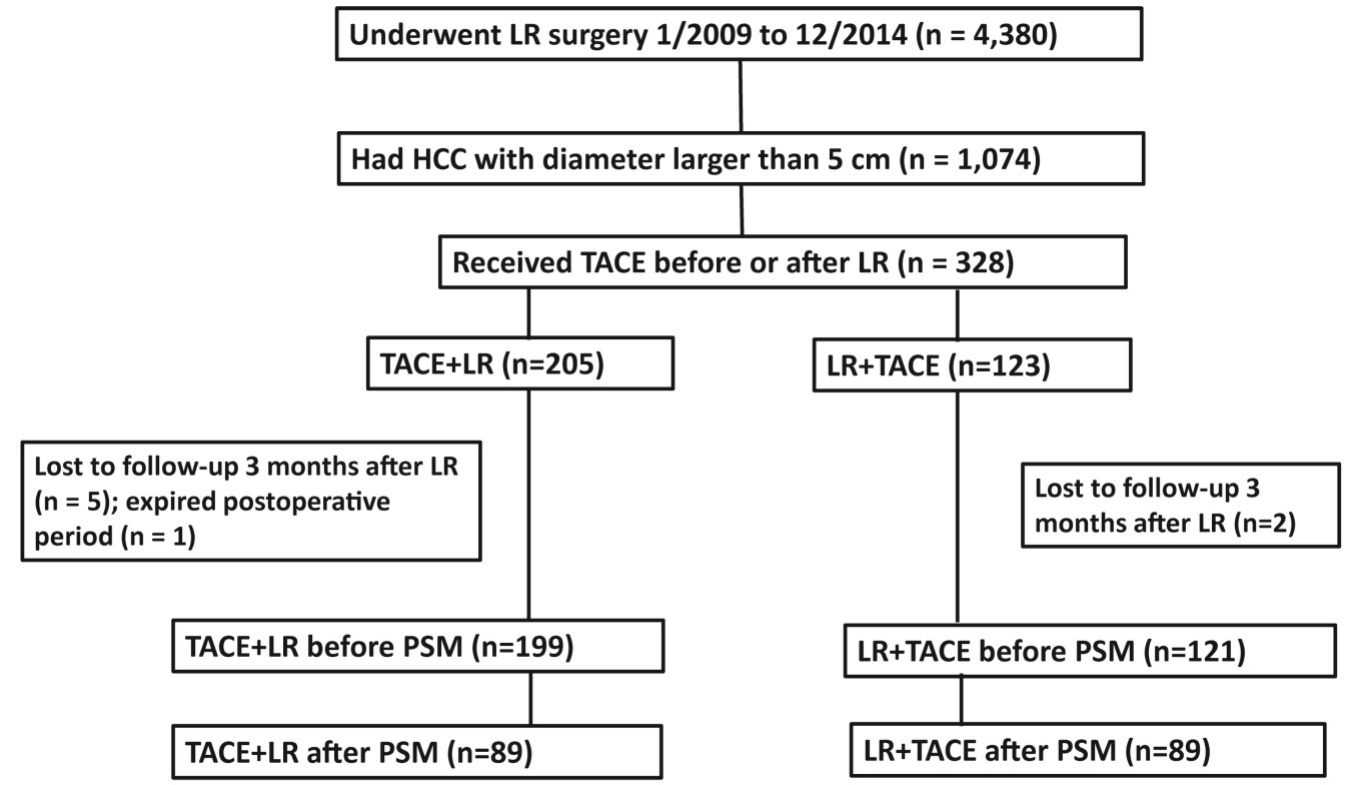
Flowchart of patient selection. Abbreviations: LR, liver resection; HCC, hepatocellular carcinoma; TACE, transarterial chemoembolization; PSM, propensity score matching.

For the study, we divided the analytic cohort of patients into 2 groups (Figure 1). Those receiving preoperative TACE followed by LR were assigned to the TACE + LR group, and those receiving LR followed by adjuvant TACE were allocated to the LR + TACE group. The multidisciplinary team of physicians made the decisions about whether to utilize TACE and when to administer it. In general, TACE was offered if a curative resection might not be feasible or if the risk of recurrence was high. If preoperative TACE was performed, one time of TACE was done within one month before LR. For patients accepting the repeated TACE, the last TACE was performed within one month before LR. For postoperative TACE was performed, the fist TACE was done within 3 months after LR, provided that there was no evidence of recurrence on the CT or MRI. For some patients, a second or third TACE was undertaken if the tumor response appeared incomplete or if the risk of recurrence remained high.

This study was conducted in accordance with the Declaration of Helsinki and approved by the Ethics Committee of the Sun Yat-sen University Cancer Center (approval number: B2020-038-01, March 23, 2020).The need for patient consent was waived by the ethics committee due to the retrospective nature of the study.

### Data characteristics and definitions

We collected data for each patient about their demographic and clinicopathological characteristics, including sex, age, body mass index (BMI), tumor diameter, tumor number, macrovascular invasion (imaging data), microvascular invasion, hepatitis, cirrhosis, preoperative portal hypertension (defined as esophageal varices and/or splenomegaly on imaging data, combined with a decreased platelet count [100 × 10^3^/μL or less]), intraoperative blood loss, intraoperative blood transfusion, number of TACE treatments, preoperative blood testing (including AFP, biochemical liver and renal function tests, prothrombin time [PT], and complete blood count). The platelet to lymphocyte ratio (PLR) was obtained by dividing the platelet count by the neutrophil count. The neutrophil to lymphocyte ratio (NLR) was obtained by dividing the neutrophil count by the lymphocyte count.

Overall survival (OS) was the primary endpoint for the study, and recurrence-free survival (RFS) was the secondary endpoint. OS was defined as the time from the first treatment (TACE or LR) to death or last follow-up, and RFS was defined as the time from the date of LR to tumor recurrence, death, or last follow-up (whichever came first). In addition, we defined the risk of recurrence for each patient based on preoperative imaging data and postoperative pathology. Preoperative macrovascular invasion evaluated by imaging data, and postoperative macrovascular invasion evaluated by imaging data combined with pathology. Patients were considered to be at intermediate risk of recurrence if they had a single tumor larger than 5 cm, without microvascular invasion (MVI) or macrovascular invasion. They were considered to be at high risk of recurrence if they had a single tumor larger than 5 cm with microvascular invasion or macrovascular invasion, or if they had multiple tumors with at least one tumor larger than 5 cm 18, 19.

### TACE technique

TACE was performed using digital subtraction angiography guidance through the left or right hepatic artery, or directly through a tumor-feeding arterial branch when technically feasible. Hepatic artery angiography, which was performed using a 5 Fr catheter (RH or Yashiro), was used to assess the number, sizes, locations, and blood supply of target tumors. The embolization emulsion was a mixture of Epirubicin (Farmorubicin; Pharmacia, Tokyo, Japan) 30 mg to 60 mg, Lobaplatin (Chang'an International Pharmaceutical, Hainan, China) 30 mg to 50 mg, and Lipiodol (Laboratorie Guerbet, Aulnay-sous-Bois, France) 10 mL to 30 mL, and it was infused into tumor-feeding arteries via a 2.7/2.8 Fr micro-catheter. The doses of the agents contained in the embolization emulsion were selected based on patient age, weight, comorbidity, tumor size, tumor number, and anticipated tolerance. The endpoint of the TACE procedure was reached when there was no flow in the tumor-feeding vessels.

### Liver resection

Liver resection was performed by experienced surgeons. We developed a surgical plan based on tumor size, tumor location and liver function. The hepatectomy method contains anatomical resection and non-anatomical resection, and the extent was defined using the Brisbane 2000 Terminology of Liver Anatomy and Resections 20. We applied Pringle's maneuver with cycles of clamping and unclamping times of 1 to 10 and 5 min each time, respectively, and controlled central venous pressure below 4 mmHg during parenchyma dissection to control intraoperative bleeding. Curative resection was defined as the complete removal all tumors with clear margin confirmed by histopathology.

### Follow-up

Follow-up visits were conducted at 2- or 3-month intervals for the first 18 months after LR, at 3- or 4-month intervals for the next 18 months, and at 3- or 6-month intervals thereafter. Each follow-up consisted of a physical examination, serum AFP, liver function and imaging examination (abdominal ultrasound, contrast-enhanced CT/MRI). If ultrasonography showed a new lesion but the AFP level was normal, contrast-enhanced ultrasonography and CT/MRI were conducted for confirmation. If two imaging findings indicated HCC, it was defined as a recurrence. Treatment options for patients with tumor recurrence included TACE, local ablation, repeat LR, sorafenib, or supportive therapy. The median follow-up period was 37 (range, 7-120) months and the date of last follow-up was September 30, 2019.

### Statistics

Clinical and pathological characteristics were summarized using means with standard deviations (SD) for continuous covariates and frequencies with proportions for categorical covariates. Continuous variables were compared using the Mann-Whitney U test and categorical variables were compared using either Pearson's χ2 test or Fisher's exact test. OS and RFS curves were estimated using the Kaplan-Meier method and compared by log-rank test. Univariate and multivariate Cox regression analyses were used to investigate the impact of potential prognostic factors on recurrence and death, including the impact of TACE pre- vs. post-LR on RFS and OS. Variables identified as significant in univariate analysis were entered into the multivariate Cox proportional hazards regression analysis. Regression results were reported as hazard ratios (HR) with 95% confidence intervals (CI), and they were estimated using the nonparametric log-rank test. All comparisons were two sided. SPSS 22.0 software (IBM Corp., Armonk, NY, USA) was used for all statistical analyses. *P*<0.05 was considered statistically significant.

### Propensity score matching (PSM)

A PSM method for creating clinically comparable cohorts was used to balance the potential biases between two groups. The propensity score was estimated using a multivariate logistic regression by using variables of sex, age, BMI, tumor diameter, tumor number, macrovascular invasion, hepatitis, portal hypertension, AFP, platelet, cirrhosis, albumin, alanine aminotransferase, blood loss, blood transfusion, microvascular invasion, and number of TACE treatments. Patients were matched 1:1 using the nearest neighbor method with a caliber of 0.05; the matching process has been described in a previous study 10.

## Results

### Study groups

We identified 4,380 patients with HCC who were treated with LR during the study period, of whom 1,074 had HCC tumors larger than 5cm (Figure 1). Initially, 328 patients met the criteria for our study. Among the 328 patients, 205 had TACE performed before LR and 123 had TACE performed after LR. However, 7 of these patients were lost to follow-up 3 months after LR and 1 patient expired during the postoperative period. This resulted in 199 patients in the TACE + LR group and 121 patients in the LR + TACE group. The 199 patients in the TACE + LR group received a total of 243 TACE treatments, and 39 patients underwent multiple TACE treatments. The 121 patients in the LR + TACE group received a total of 139 TACE treatments, and 18 underwent multiple TACE treatments.

### Demographic and clinicopathological characteristics (before PSM)

The median age of all 320 patients was 48 (range, 18 to 75) years, and 285 (89.1%) of these patients were male. Also, 113 (35.3%) of the patients had tumor diameter of 10 cm or larger, 95 (29.7%) had multiple tumors, 64 (20.0%) had macrovascular invasion, 33 (10.3%) had comorbidity, 256 (80.0%) had hepatitis, 25 (7.8%) had portal hypertension, and 159 (49.7%) had cirrhosis (Table 1).

### Comparison of Groups

Before PSM was applied, when compared to the LR + TACE group, a significantly smaller proportion of the TACE + LR group had AFP levels greater than 400 ng/mL (40.7% vs. 61.2%, *P* < 0.001), high risk of recurrence (57.8% vs. 74.4%, *P* = 0.003), and microvascular invasion (26.6% vs. 45.9%, *P* < 0.001); The TACE + LR group also had lower mean (± SD) albumin (40.2 ± 6.8 g/L vs. 48.1 ± 53.7 g/L,* P =* 0.04) and platelet (181 ± 74 x 10^9^/L vs. 200 ± 78 x 10^9^/L, *P* = 0.03) levels (Table 1). Conversely, a significantly larger proportion of the TACE + LR group had multiple tumors (34.2% vs. 22.3%,* P =* 0.02) and hepatitis (87.4% vs. 67.2%, *P* < 0.001); There were no significant differences between the 2 groups for other tumor-related characteristics, including tumor diameter and macrovascular invasion, or for prognosis-related characteristics, including comorbidity, portal hypertension, cirrhosis, and mean intraoperative blood loss.

### Overall Survival (OS)

**Figure 2:**
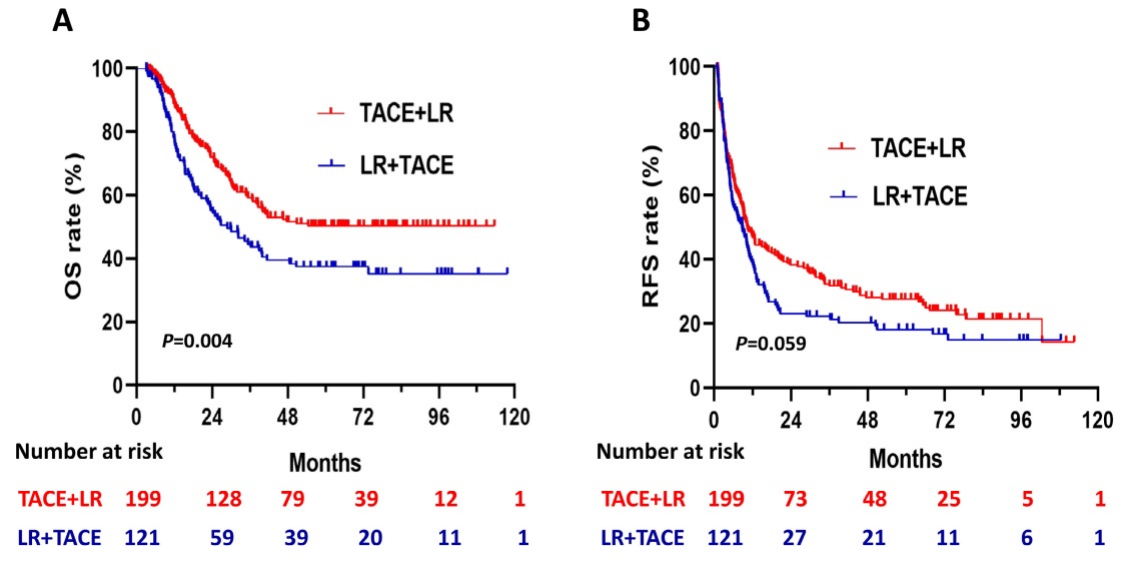
Kaplan-Meier curves for (A) overall survival (OS) and (B) recurrence-free survival (RFS) in patients with hepatocellular carcinoma (HCC) larger than 5 cm, comparing those who underwent transhepatic arterial chemotherapy and embolization (TACE) + LR to those who underwent LR + TACE prior to propensity score matching.

During the follow- up period, 88 (44.2%) of the patients in the TACE + LR group and 69 (57.0%) of the patients in the LR + TACE group had died. Before PSM, OS rates were significantly higher in the TACE + LR group than in the LR + TACE group at 1 year (89.0% vs. 78.2%, *P* = 0.008), 2 years (72.0% vs. 55.2%, *P* = 0.001), 3 years (59.2% vs. 44.6%, *P* = 0.008), and 5 years (50.3% vs. 37.3%, *P* = 0.01) (Table 2). Moreover, for the entire study period, the TACE + LR group exhibited significantly higher OS than the LR + TACE group (*P* = 0.004) (Figure 2A).

### Recurrence-free survival (RFS)

Before PSM, the cumulative recurrence-free survival (RFS) rates were not significantly higher in the TACE + LR group than the LR + TACE group at 1 year (47.1% vs. 39.1%, *P* = 0.08), but they were significantly higher at 2 years (38.3% vs. 23.1%, *P* = 0.003), 3 years (31.7% vs. 21.3%, *P* = 0.02), and 5 years (27.5% vs. 18.1%, *P* = 0.03) (Table 2). The late recurrence rate (RFS > 2 years) was significantly higher in the TACE + LR group than the LR + TACE group (*P* = 0.001). Conversely, the early recurrence rate (RFS ≤ 2 years) was lower in the TACE + LR group, but this difference was not significant (*P* = 0.08). The median RFS was 10.4 (95% CI, 8.0-12.8) months in the TACE + LR group and 8.8 (95% CI, 5.8-11.7) months in the LR + TACE group, a difference of 1.6 months. However, for the entire study period, the difference in RFS between the 2 groups was not statistically significant (*P* = 0.06) (Figure 2B).

### Recurrence patterns

During the follow- up period, among the 199 patients in the TACE + LR group, 148 (74.4%) had experienced a recurrence, and of the 121 patients in the LR + TACE group 98 (81.0%) had experienced a recurrence. A total of 140 of the 148 patients in the TACE + LR group and 91 of the 98 patients in the LR + TACE group had recurrence location data that we could analyze and received treatment in our department (Table 3). For the TACE + LR and the LR + TACE groups, more than half of recurrences occurred in the liver (65.7% and 68.1%, respectively), and the recurrence patterns did not differ significantly between the 2 groups (*P* = 0.82).

### Propensity score matching (PSM) analysis

**Figure 3:**
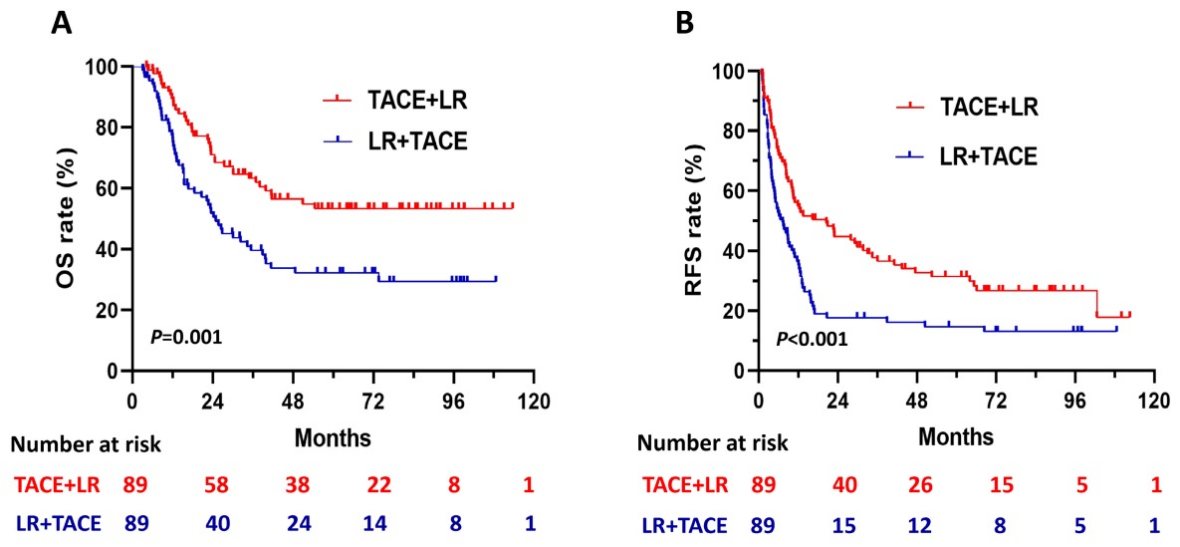
Kaplan-Meier curves for (A) overall survival (OS) and (B) recurrence-free survival (RFS) in patients with hepatocellular carcinoma (HCC) larger than 5 cm, comparing those who underwent TACE + LR to those who underwent LR + TACE after propensity score matching (PSM).

The 1:1 PSM analysis resulted in 89 patients each in the TACE + LR and LR + TACE groups (Table 1). It also resulted in the elimination of every significant demographic and clinicopathological characteristic difference observed between the 2 groups. After PSM, OS rates remained significantly higher in the TACE + LR group than in the LR + TACE group at 1 year (89.4% vs. 77.4%, *P* = 0.02), 2 years (70.9% vs. 51.9%, *P* = 0.005), 3 years (63.3% vs. 39.6%, *P* = 0.001), and 5 years (53.4 vs. 32.3%, *P* = 0.003) (Table 2), and the OS rate for the entire period was also significantly higher in the TACE + LR group (*P* = 0.001) (Figure 3A).

After PSM, cumulative RFS rates were significantly higher in the TACE + LR group than the LR + TACE group at 1 year (55.1% vs. 35.2%, *P* = 0.003), 2 years (44.8% vs. 17.6%, *P* < 0.001), 3 years (37.8% vs. 17.6%, *P* = 0.001), and 5 years (31.4% vs. 14.7%, *P* = 0.004) (Table 2). The RFS rate for the entire period was also significantly higher in the TACE + LR group (*P* < 0.001) (Figure 3B). The pattern of recurrence did not differ between the 2 groups, even after PSM (*P* = 0.34) (Table 3).

### Prognostic factors for OS and RFS

The Cox-proportional hazard model was applied to the group of all patients after PSM, in order to assess the demographic and clinicopathological characteristics that were potentially related to poor survival or recurrence. The results showed that, macrovascular invasion (HR=3.98; 95% CI, 2.37-6.67; *P* < 0.001), microvascular invasion (HR=1.64; 95% CI, 1.04-2.58; *P* = 0.03), cirrhosis (HR=2.48; 95% CI, 1.58-3.91; *P* < 0.001), and TACE treatment after surgery (HR=1.92; 95% CI, 1.22-3.02; *P* = 0.005) were significant independent risk factors associated with poorer survival (Table 4).

Similarly, the results showed that tumor diameter (HR=1.94; 95% CI, 1.31-2.88; *P* < 0.001), tumor number (HR=1.99; 95% CI, 1.35-2.93; *P* = 0.001), macrovascular invasion (HR=3.34; 95% CI, 2.23-4.99; *P* < 0.001), microvascular invasion (HR=2.24; 95% CI, 1.54-3.25; *P* < 0.001), cirrhosis (HR=1.91; 95% CI, 1.33-2.75; *P* < 0.001), high risk of recurrence (HR=1.98; 95% CI, 1.16-3.34; *P* = 0.012), and TACE treatment after surgery (HR=1.64; 95% CI, 1.16-2.32; *P* = 0.005) were significant independent risk factors associated with tumor recurrence (Table 5).

## Discussion

Tumor recurrence after LR continues to be a substantial challenge in the clinical management of large HCC, and it is associated with poor prognosis 21. TACE is a minimally invasive therapy that has demonstrated some efficacy in the treatment of large HCC 22. TACE inhibits tumor growth by blocking tumor blood supply and creating hypoxia which induces tumor necrosis. A number of studies have shown that TACE can improve survival outcomes in patients with unresectable HCC 23-25. Other studies have also demonstrated that patients with large HCC can benefit from preoperative TACE 26, 27. On the other hand, previous studies have suggested that postoperative TACE can improve the prognosis of patients with a high risk of HCC recurrence after LR 14, 28. However, whether preoperative or postoperative TACE is more effective in prolonging survival in patients with large HCC who undergo LR is still unknown. In this study, we found that the OS rates for patients who had TACE before LR were significantly better than for those who had TACE after LR. Furthermore, a comparison of the results in our patients who received TACE prior to LR with the patients in other studies who received only LR suggests a potential benefit from the addition of TACE prior to LR 29, 30. We also found that the RFS rates for patients who had TACE before LR were better than those who had TACE after LR. This suggests that the addition of TACE prior to LR may possibly provide some protection against recurrence of HCC in this patient population.

Considering there are several clinicopathological characteristics that differed significantly between the 2 groups in our study, we applied PSM to the groups and repeated the comparisons, with the goal of limiting the bias inherent in the groups being different. However, even after PSM, the cumulative OS and RFS rates for the entire study period were also significantly higher in the TACE + LR group than in the LR + TACE group. In addition, the administration of preoperative TACE, as opposed to postoperative TACE, resulted in OS rates that were 12 percentage points higher at 1 year and 19 percentage points higher at both 2 years and 5 years. Moreover, again after applying PSM, the use of TACE prior to rather than after LR resulted in RFS rates that were 20 percentage points higher at 1 year, 27 percentage points higher at 2 years, and 16 percentage points higher at 5 years. Therefore, even with PSM, our results show both a statistically significant and a clinically sizable relative benefit of preoperative TACE in patients with large HCC.

Using of TACE either before or after LR has the potential to lower the risk of tumor recurrence and improve the survival rates of patients with large HCC 10, 31. A meta-analysis systematically reviewed the published articles of preoperative and postoperative TACE for curative resection of HCC and sought to evaluate the outcomes of the two therapies, the analysis concluded that postoperative TACE offers potential benefits for curative resection of HCC when the mean tumor size is bigger than 5 cm. However, preoperative TACE showed no evidence in improving the RFS and OS in the patients 32. Our result was in contrary with the meta-analysis above. Even though there were significant improvements for RFS and OS in the postoperative TACE compared with preoperative TACE in that meta-analysis, there was no strict clinical research to compared the between preoperative and postoperative TACE in improving the outcomes of large HCC. In theory, postoperative TACE improve the outcomes of patients with large HCC, over LR alone, by destroying residual occult intrahepatic disease close to the tumor bed or other adjacent satellite lesions not identified by perioperative imaging or during surgery 18, 33. However, TACE given after LR, which has already involved the removal of a large volume of liver tissue, may accelerate the deterioration of liver function, contribute to the suppression of host immunity against tumor progression, and negatively impact the regeneration of hepatocytes 34, 35. Collectively, these processes potentially explain the inferior long-term survival rates in the patients in our study who received TACE after LR.

Some have suggested that TACE done prior to LR might result in more difficult surgery and a higher risk for intraoperative bleeding, due to the hepatic inflammation caused by TACE 36. Conversely, others have reported that preoperative TACE seems to have little influence on subsequent surgery 10, 37. In our study, before PSM, the TACE + LR group did indeed have higher mean intraoperative blood loss and blood transfusion volumes than the LR + TACE group, while after PSM, neither of these differences was statistically significant. Thus, although we observed numerically higher volumes of blood loss and transfusion when LR was preceded by TACE, these differences were not statistically significant, nor were they likely clinically relevant.

In the present study, before PSM, we found that the proportion of patients with microvascular invasion identified pathology after surgery was significantly lower in the TACE + LR group than in the LR + TACE group. This is not surprising, and others have reported that preoperative TACE for HCC can induce massive necrosis, which can markedly reduce the amount of microvascular invasion in the tumor 10. Considering that large HCC is often associated with extensive microvascular invasion, and that microvascular invasion is a known risk factor of poor prognosis in large HCC, the relative reduction of microvascular invasion in patients receiving TACE prior to LR may provide one possible explanation for why those patients had better outcomes in our study 38. Patients with large HCC also often have micrometastases, which traditional imaging modalities usually fail to detect 39. Preoperative TACE may be able to find them and may be effective in shortening the development of micrometastases, by interrupting the process of local tumor microvascular invasion at an earlier point in time. This may be another possible explanation for the better long-term outcomes in patients in our study who received preoperative TACE.

In the population of our study, the significant independent predictors of poorer overall survival were macrovascular invasion, microvascular invasion, cirrhosis, and TACE treatment after surgery. The significant independent predictors of HCC recurrence were tumor diameter, multiple tumors, macrovascular invasion, microvascular invasion, cirrhosis, and TACE treatment after surgery. These findings are similar to those in other studies 10, 18. Additionally, patients in our study having multiple TACE treatments, either before or after LR, had RFS and OS rates that did not differ significantly from the rates of those who had only a single TACE treatment. Others have reported that a single TACE treatment (which includes hepatic angiography) generally allows for the detection of any additional small HCC satellite nodules and the confirmation that local tumor vascular supply has been disrupted 36, 40. They concluded that preoperative TACE should not be repeated and that doing so would have no significant effect on recurrence and survival. The results from our study suggest that patients with large HCC need only be treated with a single TACE treatment for maximum benefit.

This study has several limitations. First, it was a retrospective single-center study. A prospective, randomized study involving multiple centers and a more heterogeneous patient population would be necessary to confirm our findings. Second, patients in this study were not specifically evaluated for whether they had a good response to TACE prior to LR. Future studies should incorporate radiomics or biological markers to help measure the response to TACE, so that only those patients who have a good response are included in comparisons. Third, measuring the response to TACE would also be helpful clinically, because patients who did not have a good response could then be offered alternative treatments prior to LR, such as thermal ablation.

## Conclusions

Patients with large HCC undergoing LR appear to derive greater disease control and survival benefit from preoperative TACE than from postoperative TACE. A single TACE treatment appears to be sufficient; RFS and OS are not improved by the administration of multiple TACE treatments. These results provide valuable information to guide the management of patients with large HCC.

## Figures and Tables

**Table 1:** Baseline characteristics of patients who underwent TACE + LR or LR + TACE for large hepatocellular carcinoma (HCC) before and after propensity score matching^a^

Characteristics	Before Matching				After Matching (1:1)		
	TACE + LR (n=199)	LR + TACE (n=121)	*P* value		TACE + LR (n=89)	LR + TACE (n=89)	*P* value
**Sex**							
Female	25 (12.6)	10 (8.3)		0.23	14 (15.7)	9 (10.1)	0.26
Male	174 (87.4)	111 (91.7)			75 (84.3)	80 (89.9)	
**Age**, years							
< 60	158 (79.4)	98 (81.0)		0.73	73 (82.0)	70 (78.7)	0.57
≥ 60	41 (20.6)	23 (19.0)			16 (18.0)	19 (12.3)	
**Child-Pugh grade^b^**							
A	190 (95.5)	117 (96.7)		0.59	86 (96.6)	87 (97.8)	0.65
B	9 (4.5)	4 (3.3)			3 (3.4)	2 (2.2)	
**Tumor diameter**, cm							
< 10	121 (60.8)	86 (70.5)		0.06	63 (70.8)	57 (64)	0.34
≥ 10	78 (39.2)	35 (29.5)			26 (29.2)	32 (36)	
**Tumor number**							
Single	131 (65.8)	94 (77.7)		0.02	65 (73.0)	63 (70.8)	0.73
Multiple	68 (34.2)	27 (22.3)			24 (27.0)	26 (29.2)	
**Macrovascular invasion^c^**	35 (17.6)	28 (23.0)		0.23	19 (21.3)	26 (29.2)	0.23
**Comorbidity^d^**	24 (12.1)	9 (7.4)		0.19	5 (5.6)	7 (7.9)	0.55
**Hepatitis^e^**	174 (87.4)	82 (67.2)		<0.001	67 (75.3)	66 (74.2)	1.00
**Portal hypertension^f^**	14 (7.0)	11 (9.1)		0.51	12 (13.5)	9 (10.1)	0.49
**Alpha-fetoprotein**, ng/mL							
≤ 400	118 (59.3)	47 (38.8)		<0.001	46 (51.7)	36 (40.4)	0.13
> 400	81 (40.7)	74 (61.2)			43 (48.3)	53 (59.6)	
**Neutrophil-Lymphocyte Ratio**							
≤ 2.8	99 (49.7)	61 (50.4)		0.91	45 (50.6)	46 (51.7)	0.88
> 2.8	100 (50.3)	60 (49.6)			44 (49.4)	43 (48.3)	
**Platelet-Lymphocyte Ratio**							
≤ 97	67 (33.7)	43 (35.5)		0.73	29 (32.6)	35 (39.3)	0.35
> 97	132 (66.3)	78 (64.5)			60 (67.4)	54 (60.7)	
**Risk of recurrence^g^**							
Intermediate	84 (42.2)	31 (25.6)		0.003	37 (41.6)	26 (29.2)	0.08
High	115 (57.8)	90 (74.4)			52 (58.4)	63 (70.8)	
**Microvascular invasion^h^**	53 (26.6)	56 (45.9)		<0.001	23 (25.8)	31 (34.8)	0.19
**Cirrhosis^h^**	102 (51.3)	57 (47.1)		0.47	40 (44.9)	31 (34.8)	0.17
**Number of TACE treatments**							
Single	160 (80.4)	103 (85.1)		0.28	74 (83.1)	80 (89.9)	0.40
Multiple	39 (19.6)	18 (14.9)			14 (16.9)	9 (10.1)	
**Body Mass Index**, kg/m^2^	22.3±3.2	22.3±2.7		0.68	21.9±3.4	22.1±2.7	0.83
**Prothrombin time**, seconds	12.1±1.3	12.03±1.1		0.57	12.1±1.1	11.9±1.1	0.58
**Alanine aminotransferase**, U/L	77.4±149.1	62.9±79.9		0.32	75.3±96.7	67.9±90.3	0.51
**Albumin**, g/L	40.2±6.7	48.0±53.7		0.04	40.6±8.1	45.8±45.6	0.30
**Total Bilirubin**, μmol/L	14.2±7.2	14.3±7.8		0.73	15.1±7.7	14.07±7.3	0.38
**Platelet count**, 10^9^/L	181±74	200±78		0.03	191±67	184±67	0.52
**White Blood Count**, 10^9^/L	6.59±2.81	6.96±2.28		0.22	6.39±2.01	6.79±2.21	0.21
**Blood loss**, mL	748±742	622±599		0.11	802±868	680±670	0.30
**Blood transfusion**, mL	251±458	146.±299		0.03	256±444	169±330	0.14
							

Data are n (%) or mean and standard deviation. ^a^ A propensity score matching (PSM) method for creating clinically comparable cohorts was used to correct for potential biases. Patients were matched 1:1 using the nearest neighbor method with a caliber of 0.05. ^b^ Child-Pugh grade is a measure of severity of liver function, based on 5 clinical factors: PT or INR, albumin, bilirubin, ascites, and hepatic encephalopathy. ^c^ Macrovascular tumor thrombus defined as tumor located in the intrahepatic branches of the portal or hepatic veins. ^d^ Comorbidity defined as hypertension, diabetes, coronary disease, and/or severe anemia. ^e^ Hepatitis defined preoperatively as a history of chronic HBV infection and/or positive hepatitis C virus RNA test. ^f^ Portal hypertension defined preoperatively as esophageal varices and/or splenomegaly on imaging studies combined with a decreased platelet count [100 × 10^3^/μL or less]). ^g^ Risk of recurrence defined as intermediate if patient had a single tumor larger than 5 cm, without microvascular invasion or macrovascular tumor thrombus, and defined as if patient had a single tumor larger than 5 cm, with microvascular invasion or macrovascular tumor thrombus, or if they had multiple tumors with at least one tumor larger than 5 cm 18. ^h^ Histopathological findings from liver resection (LR) specimen. Abbreviations: TACE, transarterial chemoembolization; LR, liver resection.

**Table 2:** Overall survival (OS) and recurrence-free survival (RFS) of patients who underwent TACE + LR or LR + TACE for large hepatocellular carcinoma (HCC) before and after propensity score matching^a^

Variables	Before Matching			After Matching (1:1)		
	TACE + LR (n=199) %	LR + TACE (n=121) %	*P* value	TACE + LR (n=89) %	LR + TACE (n=89) %	*P* value
**1-year OS**	89.0	78.2	0.008	89.4	77.4	0.02
**2-year OS**	72.0	55.2	0.001	70.9	51.9	0.005
**3-year OS**	59.2	44.6	0.008	63.3	39.6	0.001
**5-year OS**	50.3	37.3	0.01	53.4	32.3	0.003
**1-year RFS**	47.1	39.1	0.08	55.1	35.2	0.003
**2-year RFS**	38.3	23.1	0.003	44.8	17.6	<0.001
**3-year RFS**	31.7	21.3	0.02	37.8	17.6	0.001
**5-year RFS**	27.5	18.1	0.03	31.4	14.7	0.004

^a^ A propensity score matching (PSM) method for creating clinically comparable cohorts was used to correct for potential biases. Patients were matched 1:1 using the nearest neighbor method with a caliber of 0.05. Abbreviations: TACE, transarterial chemoembolization; LR, liver resection. OS, overall survival; RFS; recurrence-free survival

**Table 3:** Recurrence patterns in patients who underwent TACE + LR or LR + TACE for large hepatocellular carcinoma (HCC) before and after propensity score matching^a^

Variables	Before Matching			After Matching (1:1)		
	TACE+LR (n=199)	LR +TACE (n=121)	P value	TACE+LR (n=89)	LR+ TACE (n=89)	P value
**Total recurrence**, n (%)	140 (70.4)	91 (75.2)		60 (67.4)	69 (77.5)	
**Recurrence patterns**, n (%)			0.82			0.34
Intrahepatic	92 (65.7)	62 (68.1)		34 (56.7)	45 (65.2)	
Extrahepatic	26 (18.6)	14 (15.4)		14 (23.3)	12 (17.4)	
Both	22 (15.7)	15 (16.5)		12 (20.0)	12 (17.4)	

^a^ A propensity score matching (PSM) method for creating clinically comparable cohorts was used to correct for potential biases. Patients were matched 1:1 using the nearest neighbor method with a caliber of 0.05. ^b^ A total of 148 patients in the TACE + LR group and 98 patients in the LR + TACE group experienced recurrences. Of these, 140 patients in the TACE + LR group and 91 patients in the LR + TACE group had recurrence location data and received treatment in our department, so were included in this analysis. Abbreviations: TACE, transarterial chemoembolization; LR, liver resection.

**Table 4:** Univariate and multivariate analyses for overall survival for large hepatocellular carcinoma patients who underwent a combination of TACE and liver resection after propensity score matching^a^

Characteristics	Patients in Each Covariate N : N	Univariate Analysis		Multivariate Analysis	
		HR (95% CI)	P value	HR (95% CI)	P value
**Sex** (female vs. male)	23 : 155	0.91 (0.50-1.68)	0.77		
**Age**, years (≥ 60 vs. < 60)	35 : 143	0.94 (0.56-1.57)	0.80		
**Tumor diameter** (≥ 10 vs. < 10), cm	58 : 120	1.75 (1.45-2.67)	0.009		0.42
**Tumor number** (multiple vs. single )	50 : 128	1.44 (0.92-2.25)	0.11		
**Macrovascular invasion^b^** (positive vs. negative)	45 : 143	5.80 (3.70-9.10)	<0.001	3.98 (2.37-6.67)	<0.001
**Comorbidity^c^** (yes vs. no)	12 : 166	0.49 (0.18-1.33)	0.16		
**Portal hypertension^d^** (yes vs. no)	157 : 21	1.28 (0.73-2.27)	0.39		
**Alpha-fetoprotein**, ng/mL (> 400 vs. ≤ 400)	96 : 82	1.54 (1.01-2.34)	0.046		0.72
**Neutrophil-Lymphocyte Ratio** (> 2.8 vs. ≤ 2.8 )	87 : 91	1.31 (0.87-1.99)	0.20		
**Platelet-Lymphocyte Ratio** (> 97 vs. ≤ 97)	114 : 64	0.99 (0.65-1.53)	--		
**Microvascular invasion^e^** (positive vs. negative)	54 : 124	1.64 (1.04-2.58)	0.002	1.64 (1.04-2.58)	0.03
**Hepatitis** (yes vs. no)	133:65	1.49 (0.89-2.51)	0.126		
**Cirrhosis^e^** (yes vs. no)	71: 107	3.05 (2.01-4.64)	<0.001	2.48 (1.58-3.91)	<0.001
**Number of TACE** (multiple vs. single)	26 : 152	1.03 (0.60-1.76)	0.93		
**Risk of recurrence** (high vs. intermediate)	99:79	3.65 (2.32-5.74)	<0.001	1.72 (0.92-3.12)	0.093
**Time of TACE** (post vs. pre-resection)	89 : 89	1.97 (1.30-3.00)	0.002	1.92 (1.22-3.02)	0.005

^a^ A propensity score matching (PSM) method for creating clinically comparable cohorts was used to correct for potential biases. Patients were matched 1:1 using the nearest neighbor method with a caliber of 0.05. ^b^ Macrovascular tumor thrombus defined as tumor located in the intrahepatic branches of the portal or hepatic veins. ^c^ Comorbidity defined as hypertension, diabetes, coronary disease, and/or severe anemia. ^d^ Portal hypertension defined preoperatively as esophageal varices and/or splenomegaly on imaging studies combined with a decreased platelet count [100 × 10^3^/μL or less]). ^e^ Histopathological findings from liver resection (LR) specimen. Abbreviations: TACE, transarterial chemoembolization; HR, hazard ratio; CI, confidence interval

**Table 5:** Univariate and multivariate analyses for recurrence for large hepatocellular carcinoma patients who underwent a combination of TACE and hepatic resection after propensity score matching^a^

Characteristics	Patients in each Covariate N : N	Univariate Analysis		Multivariate Analysis	
		HR (95% CI)	P value	HR (95% CI)	P value
**Sex** (female vs. male)	23 : 155	0.78 (0.47-1.33)	0.37		
**Age**, years (≥ 60 vs. < 60)	35 : 143	0.93 (0.61-1.41)	0.73		
**Tumor diameter** (≥ 10 vs. < 10), cm	58 : 120	2.62 (1.84-3.73)	<0.001	1.94 (1.31-2.88)	<0.001
**Tumor number** (multiple vs. single )	50 : 128	1.56 (1.08-2.25)	0.02	1.99 (1.35-2.93)	0.001
**Macrovascular invasion^b^** (positive vs. negative)	45 : 143	4.42 (2.99-6.55)	<0.001	3.34 (2.23-4.99)	<0.001
**Comorbidity^c^** (yes vs. no)	12 : 166	0.79 (0.40-1.56)	0.50		
**Portal hypertension^d^** (yes vs. no)	157 : 21	1.02 (0.61-1.70)	0.94		
**Alpha-fetoprotein**, ng/mL (> 400 vs. ≤ 400)	96 : 82	1.26 (0.90-1.76)	0.18		
**Neutrophil-Lymphocyte Ratio** (> 2.8 vs. ≤ 2.8 )	87 : 91	1.24 (0.89-1.73)	0.21		
**Platelet-Lymphocyte Ratio** (> 97 vs. ≤ 97)	114 : 64	0.86 (0.61-1.23)	0.41		
**Microvascular invasion^e^** (positive vs. negative)	54 : 124	2.71 (1.88-3.92)	<0.001	2.24 (1.54-3.25)	<0.001
**Hepatitis** (yes vs. no)	133:65	1.1.4 (0.78-1.68)	0.502		
**Cirrhosis^e^** (yes vs. no)	71: 107	2.05 (1.46-2.89)	<0.001	1.91 (1.33-2.75)	<0.001
**Number of TACE** (multiple vs. single)	26 : 152	0.94 (0.59-1.50)	0.79		
**Risk of recurrence** (high vs. intermediate)	99:79	4.21 (2.87-6.18)	<0.001	1.98 (1.16-3.34)	0.012
**Time of TACE** (post vs. pre-resection)	89 : 89	1.78 (1.27-2.49)	0.001	1.64 (1.16-2.32)	0.005

^a^ A propensity score matching (PSM) method for creating clinically comparable cohorts was used to correct for potential biases. Patients were matched 1:1 using the nearest neighbor method with a caliber of 0.05. ^b^ Macrovascular tumor thrombus defined as tumor located in the intrahepatic branches of the portal or hepatic veins. ^c^ Comorbidity defined as hypertension, diabetes, coronary disease, and/or severe anemia. ^d^ Portal hypertension defined preoperatively as esophageal varices and/or splenomegaly on imaging studies combined with a decreased platelet count [100 × 10^3^/μL or less]). ^e^ Histopathological findings from liver resection (LR) specimen. Abbreviations: TACE, transarterial chemoembolization; HR, hazard ratio; CI, confidence interval
